# No association between blood count levels and whole-blood cobalt and chromium levels in 1,900 patients with metal-on-metal hip arthroplasty

**DOI:** 10.1080/17453674.2020.1827191

**Published:** 2020-10-02

**Authors:** Noora Honkasaari, Olli Lainiala, Outi Laine, Aleksi Reito, Antti Eskelinen

**Affiliations:** a Coxa Hospital for Joint Replacement, Tampere; b University of Tampere, Faculty of Medicine and Life Sciences, Tampere; c Tampere University Hospital, Department of Internal Medicine, Tampere, Finland

## Abstract

**Background and purpose** — The accelerated wear of poorly functioning metal-on-metal (MoM) hip implants may cause elevated whole-blood cobalt (Co) and chromium (Cr) levels. Hematological and endocrinological changes have been described as the most sensitive adverse effects due to Co exposure. We studied whether there is an association between whole-blood Co/Cr levels and leukocyte, hemoglobin, or platelet levels.

**Patients and methods** — We analyzed whole-blood Co and Cr values and complete blood counts (including leukocytes, hemoglobin, platelets) from 1,900 patients with MoM hips. The mean age at the time of whole-blood metal ion measurements was 67 years (SD 10). The mean time from primary surgery to whole-blood metal ion measurement was 8.2 years (SD 3.0). The mean interval between postoperative blood counts and metal ion measurements was 0.2 months (SD 2.7).

**Results** — The median Co value was 1.9 µg/L (0.2–225), Cr 1.6 µg/L (0.2–125), mean leukocyte count 6.7 × 10^9^/L (SD 1.9), hemoglobin value 143 g/L (SD 13), and platelet count 277 × 10^9^/L (SD 70). We did not observe clinically significant correlations between whole-blood Co/Cr and leukocyte, hemoglobin, or platelet counts.

**Interpretation** — Elevated whole-blood Co and Cr values are unlikely to explain abnormal blood counts in patients with MoM hips and the reason for possible abnormal blood counts should be sought elsewhere.

The abnormal wear of poorly functioning MoM implants may cause elevated whole-blood cobalt (Co) and chromium (Cr) levels (Brodner et al. 2003, Cheung et al. 2016). Soft-tissue reactions termed “pseudotumors” related to poorly functioning MoM hip replacements have been widely described (Boardman et al. 2006, Gruber et al. 2007, Pandit et al. 2008). The use of MoM implants has dramatically decreased but due to their previous popularity there are still a large number of patients with MoM hip replacements under follow-up (Silverman et al. 2016).

Even though local reactions have been the most discussed, systemic reactions in patients with high-wearing hip implants have been described. Cardiomyopathy, polyneuropathy, hypothyreosis, and polycythemia have been described in some patients with MoM hip implants and in patients with fractured ceramic-on-ceramic implant revised to metal-on-polyethylene, resulting in abrasive wear of the CoCr head by ceramic fragments (Cheung et al. 2016). Systemic adverse events have been linked mostly to Co, and hematological and thyroid effects have been described as the most sensitive responses to Co in humans (Tvermoes et al. 2014). A case report described polycythemia with hemoglobin 190 g/L due to massive abrasive CoCr head wear when a ceramic-on-ceramic implant had been revised to metal-on-polyethylene after fracture of the ceramic liner (Gilbert et al. 2013). Several studies have suggested that blood lymphocyte counts may be affected by implant metals from MoM hip replacements (Hart et al. 2009, Hailer et al. 2011, Penny et al. 2013, Chen et al. 2014, Briggs et al. 2015, Markel et al. 2018). Although thrombocytopenia has not been linked to implant metals, it has been reported that platelets adhere to and are activated by CoCr (Ollivier et al. 2017).

Complete blood count including leukocyte count, hemoglobin, and platelets is among the most used blood tests in the world (Horton et al. 2019) and 10–20% of the measurements include abnormal values (Tefferi et al. 2005). Due to wide media attention to MoM hip replacements, patients with MoM hips are sometimes worried whether their abnormal laboratory findings are related to their hip replacement. We sought to find out whether whole-blood metal ion levels are associated with blood count. Our hypothesis was that if Co or Cr affected leukocytes, hemoglobin, or platelets at concentrations noted in our study group, we would observe an upward or downward trend (depending on variable) when blood Co or Cr concentrations are approaching the highest values.

## Patients and methods

This is a retrospective study. In September 2010 the United Kingdom Medicines and Healthcare Products Regulatory Agency released a medical device alert concerning MoM hip implants (MHRA 2010) and DePuy Orthopaedics recalled their ASR MoM hip replacement systems (DePuy Orthopaedics 2010). After the recall our institution started the systematic screening of all patients with Articular Surface Replacement (ASR; DePuy Orthopaedics, Warsaw, IN, USA) resurfacing or total hip arthroplasty (THA) hips. Screening included whole blood Co and Cr measurements, imaging, and clinical examination. In 2012 the screening program was expanded to include all our patients with any MoM hip implant brand, but implants other than ASRs were not systematically imaged.

For this study, we included all our patients with Co and Cr measurements and blood count drawn within an arbitrary 6-month interval. Between November 1999 and February 2012, 3,013 MoM hips were implanted in 2,520 patients at our institution. Of these patients, whole blood Co and Cr measurements were available for 2,260 patients.

### Blood Co and Cr samples

The blood samples for the Co and Cr measurements were drawn from the antecubital vein. The first 10 mL was discarded to avoid contamination by the metal ions released from the needle. The samples were sent to an independent laboratory for analysis. The measurements were performed with dynamic reaction cell inductively coupled plasma mass spectrometry (Agilent 7500 cx or 8800, Agilent Technologies, Santa Clara, CA, USA) between October 2009 and March 2018.

### Blood counts

For the complete blood counts, 3 mL of blood was drawn into an EDTA tube. Leukocyte count, hemoglobin, hematocrit, erythrocyte count, mean corpuscular volume, and mean hemoglobin concentration as well as platelet count were then analyzed. Reference values for blood count values provided by our hospital laboratory were used. The complete blood counts were measured between October 2009 and June 2018. We used the complete blood count closest to the latest Co and Cr values. As the data was not prospectively collected and the whole blood Co and Cr measurements were not performed on the same day as the blood count, we chose 6 months as an arbitrary cut-off to exclude patients with long interval between the measurements. Of the 2,260 patients with whole-blood metal ion levels available, 1,900 had their blood count measured less than 6 months before or after the whole-blood metal ion measurement and were therefore included in the study. 572 of the patients went through a revision and for them last pre-revision measurements were used. For 314 patients there was no complete blood count available within 6 months of the latest whole-blood Co and Cr values, and thus an older metal ion measurement fulfilling the condition of blood count measured within a 6-month interval was used. For 258 patients leukocyte differentials were available and they were analyzed as a subgroup.

### Statistics

As the distributions of whole-blood Co and Cr values are skewed, median values (ranges) are reported. Blood hemoglobin value, leukocyte, and platelet counts are normally distributed, so mean values (SD) are used. Spearman’s correlation coefficients were calculated to test whether there is correlation between whole-blood metal ion levels and either hemoglobin, leukocyte, or platelet counts. For Spearman’s correlation coefficient, we calculated 95% confidence intervals (CI). We chose a correlation coefficient of 0.225 (or –0.225) as the limit for clinically significant, equaling roughly 5% of variance for a dependent variable explained by an independent variable. If the observed CIs for correlation coefficients did not include 0.225 the correlation coefficient was considered as clinically not significant. We also calculated the change in blood count components for those 1,873 (99%) patients with preoperative blood counts available.

The analyses were performed for all patients regardless of the implant brand and manufacturer or whether they had unilateral or bilateral MoM hip implants even though the blood concentrations are different among them, because, when considering the systemic effects of Co and Cr, it is irrelevant whether the Co and Cr in the blood are from 1 or 2 hips and which implant brand is used.

### Ethics, funding, and potential conflicts of interest

Permission for the study was obtained from the institutional review board (permission number R17524S). This work was supported by the competitive research funds of Pirkanmaa Hospital District, Tampere, Finland, representing governmental funding. No conflicts of interest declared except for AE, who is paid lecturer for Zimmer Biomet, and receives institutional research support (not related to current study) from DePuy Synthes and Zimmer Biomet.

## Results

Of the 1,900 patients included in the study, 81% had unilateral and 19% had bilateral MoM replacement (Table 1). 58% of the patients were male. The mean age at the time of whole-blood metal ion measurement was 67 years (SD 10). The mean time between the primary surgery and metal ion measurements was 8.2 years (SD 3.0). The mean time interval between blood metal ion measurements and postoperative blood counts was 0.2 months (SD 2.7).

**Table 1.** Metal-on-metal hip implant brands of the implant included in this study

Hip resurfacing implants				Total hip implants				Total hip and resurfacing implants	
unilateral		bilateral		unilateral THA		bilateral THA			
n = 536		n = 156		n = 998		n = 189		n = 21	
ASR	198	BHR/BHR	52	ASR	278	ASR/ASR	52	BHR/BHR	6
BHR	194	ASR/ASR	52	Pinnacle	259	Pinnacle/Pinnacle	50	ReCap/BHR	3
Durom	92	Durom/Durom	9	ReCap	157	BHR/BHR	11	ASR/ASR	2
Other	52	Other	43	Other	304	Other	76	Other	10

ASR = Articular Surface Replacement (Depuy Orthopaedics, Warsaw, IN, USA);

BHR = Birmingham Hip Resurfacings (Smith & Nephew, Memphis, TN, USA);

Durom (Zimmer, Warsaw, IN, USA); Pinnacle (Depuy), ReCap (Biomet, Warsaw, IN, USA) .

The median value for whole-blood Co concentration was 1.9 µg/L (0.2–225) and for Cr 1.6 µg/L (0.2–125). Mean postoperative leukocyte count was 6.7 × 10^9^/L (SD 1.9), hemoglobin 143 g/L (SD 13), and platelet count 277 × 10^9^/L (SD 70). Mean preoperative leukocyte count was 6.8 × 10^9^/L (SD 2.5), hemoglobin 140 g/L (SD 14), and platelet count 245 × 10^9^/L (SD 71). The mean time between preoperative and last postoperative blood count was 8.3 years (SD 3.0). The mean change in leukocyte (Δleukocytes) count was 0.1 × 10^9^/L (SD 2.4), in hemoglobin (Δhemoglobin) –3.0 g/L (SD 13), and in platelet count (Δplatelets) –32 × 10^9^/L (SD 61).

Spearman’s correlation coefficients (ρ) between whole-blood Co/Cr and complete blood count parameters are presented in Table 2 (for scatter plots in Figure 1, see Supplementary data). None of the 95% CIs for ρ included predefined 0.225 or –0.225 (equaling roughly 5% of variance for the dependent variable explained by the independent variable), indicating that clinically significant associations can be excluded.

**Table 2.** Spearman’s correlation coefficients (ρ) and 95% confidence intervals among whole-blood cobalt, chromium levels, hemoglobin concentration, and leukocyte and platelet counts

Factor	Cobalt	Chromium
Leukocytes	–0.05 (–0.09 to 0.000)	–0.08 (–0.12 to –0.03)
Hemoglobin	–0.09 (–0.13 to –0.05)	–0.12 (–0.16 to –0.07)
Platelets	0.06 (0.02 to 0.11)	0.13 (0.09 to 0.18)
ΔLeukocytes	–0.05 (–0.10 to –0.01)	–0.05 (–0.09 to –0.002)
ΔHemoglobin	0.02 (–0.02 to 0.07)	0.05 (0.007 to 0.10)
ΔPlatelets	0.02 (–0.03 to 0.07)	0.05 (0.003 to 0.09)

Δ = change from preoperative to last postoperative value.

Leukocyte differentials including lymphocytes, neutrophils, eosinophils, basophils, and monocytes were available for 258 patients. This subgroup included 53% men. The mean age at the time of blood metal ion measurement was 67 years (SD 10), mean time from primary surgery to metal ion measurement was 7.8 years (SD 2.7), and interval between metal ion and leukocyte differentials 0.9 months (SD 3.0). The Spearman’s correlation coefficients (ρ) between whole-blood Co and blood eosinophil count (0.11, CI –0.01 to 0.23) and between Cr and monocytes count (–0.08, CI –0.20 to 0.42) were the only ρ between Co/Cr and leukocyte differential counts in which 0.225 was not excluded from 95% CI (Table 3, Figure 2, see Supplementary data). For all other analyses, clinically significant associations were excluded.

**Table 3.** Spearman’s correlation coefficients (ρ) and 95% confidence intervals among whole-blood cobalt, chromium levels, and leukocyte differentials

Factor	Cobalt	Chromium
Lymphocytes	–0.09 (–0.21 to 0.03)	–0.09 (–0.21 to 0.03)
Neutrophils	–0.05 (–0.17 to 0.07)	–0.07 (–0.19 to 0.06)
Eosinophils	0.11 (–0.01 to 0.23) ^a^	0.05 (–0.07 to 0.17)
Basophils	–0.02 (–0.14 to 0.11)	0.003 (–0.12 to 0.13)
Monocytes	–0.06 (–0.18 to 0.06)	–0.08 (–0.20 to 0.42) ^a^

**^a^**95% confidence interval of the Spearman correlation coefficient includes value of 0.225, indicating that 5% variance for blood eosinphils explained by whole blood cobalt concentration, and 5% variance for blood monocytes explained by whole blood chromium concentrations cannot be ruled out by this analysis.

## Discussion

As many factors affect the blood leukocyte, hemoglobin, and platelet values, for this study we chose a rather low ρ of 0.225/–0.225 to represent clinically significant correlation. This ρ equals roughly 5% of variance for a dependent variable explained by an independent variable. Despite choosing a low ρ-value to represent clinical significance, none of the CIs for correlation coefficients among whole-blood Co/Cr and leukocytes, hemoglobin, or platelet values included that value, excluding a clinically significant association. Therefore, the exposure to elevated whole-blood Co and Cr levels seems unlikely to explain clinically noticeable or relevant changes in complete blood counts of patients with MoM hip and blood Co/Cr levels comparable to those in this study. Consequently, the cause for abnormal complete blood counts should be primarily sought elsewhere. Of the leukocyte differential counts, only the CIs for ρ between whole-blood Co and blood eosinophils and between blood Cr and monocytes included 0.225. For other analyses, clinically significant association was excluded.

There are some limitations in our study. 1st, we examined only the association between whole-blood metal ion levels and patients’ blood count, the study setting limits us from evaluating causality. 2nd, we defined clinically significant correlation as correlation coefficient of 0.225 or higher. To our knowledge, there are no predefined limits for clinical significance of correlation between blood metal ions and blood counts reported in the literature. ρ of 0.225 equals roughly 5% of variance for a dependent variable explained by an independent variable. We consider 5% a rather low value, but as blood counts are affected by countless other factors we did not want to choose too high a limit. 3rd, we had no information on the patients’ comorbidities or treatments, e.g., hematologic malignancies, HIV, chemotherapy, or radiation therapy that could have affected their blood counts. Further, as this was a retrospective study the whole-blood metal ion levels and blood counts were not measured on the same date. Hematological abnormalities caused by Co are likely to be reversible (Leyssens et al. 2017) and may even normalize if exposure is removed. However, the changes in whole-blood Co and Cr levels have been mostly less than 5 µg/L at 1-year follow-up with only a few exceptions, even in our cohort of high-risk ASR implants (Reito et al. 2014, 2016). All measurements included were drawn before revision surgery (if performed), as revision may result in a radical decrease of Co and Cr levels. Furthermore, we measured whole-blood levels, which have been described as more stable compared with serum and therefore offer a better indicator of long-term cobalt exposure (Paustenbach et al. 2014). Even though measurements within a 6-month time interval were included, an SD of 2.7 months indicates that more than two-thirds of measurements were within 3 months. We believe that the interval between measurements does not prevent us from drawing conclusions.

A variety of symptoms possibly relating to Co and Cr have been described at case report level in patients with hip arthroplasties (Leyssens et al. 2017). The most devastating wear and systemic adverse events leading to death have been described resulting from ceramic fragments of a fractured ceramic implant causing abrasive third-body wear to a metal head implanted in revision (Gilbert et al. 2013, Fox et al. 2016, Peters et al. 2017). Systemic adverse events in patients with hip implants have been mainly linked to Co, which was the main ion of interest in our study (Finley et al. 2012). Exposure to Cr(VI) (hexavalent chromium) may cause hemoglobin, leukocyte, and platelet abnormalities (Ray 2016). However, Cr in blood of patients with hip implants is thought to be not Cr(VI) but less toxic Cr(III) (trivalent chromium), which is not likely to cause health risk to patients (Finley et al. 2017). Cr at higher valences (most likely Cr[VI]) has been reported in postmortem samples of 2 diabetics, suggesting that re-oxidation of Cr(III) to more toxic Cr (VI) might be possible under certain circumstances (Swiatkowska et al. 2018). We are not aware of further studies confirming or annulling this finding.

There is a case report concerning polycythemia (Hb 190 g/L) in a patient with an intensively worn CoCr head due to ceramic fragments from the previous fractured implant embedded in a new polyethylene liner resulting in extremely high (1,085 µg/L) serum Co levels (Gilbert et al. 2013). We are not aware of other studies regarding the association between Co and hemoglobin in patients with hip replacements. From the 1950s to 1980s Co was used in the treatment of anemia due to its potential to stimulate erythropoietin production, and polycythemia was described resulting from ingestion of Co. Among the published literature, Co doses resulting in blood Co concentrations of approximately 300 µg/L or less have not been associated with hematological responses, while blood Co concentrations of approximately 600 µg/L and higher have consistently been associated with polycythemia and increased hemoglobin content (Finley et al. 2012). Our findings from a hip arthroplasty population support the findings from dietary Co supplementation studies (Finley et al. 2012), as we found no tendency for polycythemia in patients with blood Co up to 225 µg/L.

Several studies have reported associations between implant metals and leukocytes, mainly concerning lymphocytes. A randomized trial including 41 patients with MoM (mean Co 0.9 µg/L and Cr 1.1 µg/L) and 44 with metal-on-polyethylene (MoP) arthroplasty reported a higher percentage of HLA DR + CD8+ T-cells and lower percentage of B-cells in the MoM group (Hailer et al. 2011). A cross-sectional study including 106 patients with MoM hip (median Co 1.7 µg/L and 2.5 µg/L for unilaterals and bilaterals, and Cr 2.3 µg/L and 2.4 µg/L, respectively) and 58 with non-MoM hip observed a reduction in the number of T-lymphocytes and B-lymphocytes in MoM patients (Hart et al. 2009). The finding was supported by a smaller randomized trial including 19 ASR hip resurfacing patients (median Co 1.4 µg/L and Cr 1.3 µg/L) and 19 patients with MoP implant (Penny et al. 2013). A study with 32 patients with MoM implant (mean serum Co 0.5µg/L and Cr 0.2 µg/L), 32 with MoP implant, and 32 controls pointed out that, in particular, CD3+, CD4+, and CD8+ T-lymphocytes were reduced in patients with MoM. In vitro studies have further characterized the reduction of lymphocyte viability and the increase in apoptosis due to implant metals (Akbar et al. 2011, Posada et al. 2015).

Most of the studies cited have described changes in only certain subpopulations of lymphocytes. Some of the findings may be random variation interpreted as actual associations, but it is possible that Co and/or Cr may have a depressive effect on certain lymphocyte subpopulations even at lower metal ion levels. Our study did not include lymphocyte subpopulations as they are not routinely measured in clinical work. This study provides information only on total leukocytes and leukocyte differentials, and possible changes in a few lymphocyte subpopulations are unlikely to affect those. We did not observe an association between whole-blood Co/Cr and blood leukocyte, hemoglobin, or platelet counts. Of the leukocyte differential counts, the only correlations that included the predefined limit of 0.225 were those between whole-blood Co and blood eosinophils, and whole-blood Cr and blood monocytes. The CIs for these correlations were rather wide. The upper limits for CI of these correlations were 0.229 (equaling roughly 5.2% of variance explained) and 0.42 (equaling 17%). The reference values for blood eosinophils at our laboratory are 0.01–0.45 × 10^9^/L and for monocytes 0.2–0.8 × 10^9^/L. Of the upper limits of reference values, the 5.2% is 0.02 × 10^9^/L and 17% is 0.1 × 10^9^/L, both of which are negligible figures as absolute concentrations. Therefore, despite the CI including the predefined limit of 0.225, these findings are unlikely to be clinically relevant. Further, as a leukocyte differential was not drawn from all patients, there is a risk for selection bias, and therefore the interpretation of this subgroup analysis has to be made with even more caution.

To our knowledge there are no case reports or studies concerning abnormal platelet values that would have been linked to implant metals. An in vitro study concerning CoCr stent materials stated that platelets adhere to and are activated by CoCr (Ollivier et al. 2017). 1 case report presented a patient with thrombocytopenia and anemia after orally ingesting a large amount of chromium picolinate (including trivalent Cr, resulting in peak Cr of 6.5 µg/L) used to enhance weight loss and to improve glycemic control (Cerulli et al. 1998). Thrombocytopenia has also been described after ingestion of chromium acid (including hexavalent Cr) mixed in a drink with homicidal intentions (Quaiser et al. 2014). We did not observe an association between implant metals and platelet count.

The problem of determining whether the new abnormalities in patients’ blood counts are related to their MoM hip implants is not an everyday problem at an orthopedic outpatient clinic. Because of patients’ worries due to massive media attention (Cohen 2011) or legal aspects of the problems with MoM hip replacements (Dyer 2010, 2018), orthopedic surgeons may occasionally come across patients asking whether some of their symptoms or laboratory findings could be related to their MoM hip replacement. Our study adds, to the existing literature, a large volume of single-center clinical data concerning the relationship between implant metals and complete blood count values with whole-blood Co concentrations up to 225 µg/L and Cr up to 125 µg/L. As our study included a large number of patients some of whom had abnormally high whole-blood Co and Cr ion concentrations, we believe this study had a good chance to reveal possible adverse hematological effects if those were to develop at concentrations included in this study. Our study is derived from a primary hospital treating patients from the municipality, regardless of their age or socioeconomic status, which makes our results generalizable.

### ^Conclusion^

In this, one of the largest single-center MoM cohorts in the world, we found no evidence of clinically significant correlations between whole-blood Co and Cr levels and leukocytes, hemoglobin, and platelets. Based on previous literature, hematological adverse effects are unlikely at Co concentrations of 300 µg/L and below, and Cr in blood of patients with hip implants is in less toxic trivalent Cr(III) form, which does not pose a health risk to patients. Our results supplement the previous results with clinical data from a hip arthroplasty population. The reasons for possible abnormal blood counts in patients with MoM hip and their Co and Cr below several hundred µg/L should be primarily sought elsewhere.  

## Supplementary Material

Supplemental Material

## Supplementary data

Figures 1 and 2 are available as supplementary data in the online version of this article, http://dx.doi.org/10.1080/17453674. 2020.1827191

The authors would like to thank Ella Lehto RN, and Heli Kupari RN, Coxa Hospital for Joint Replacement, Tampere, Finland, for maintaining the study database. They thank Reija Autio Dr. Tech., University of Tampere, Faculty of Medicine and Life Sciences, Tampere, Finland, for statistical assistance.
