# Supplementary material for: No association between blood count levels and whole-blood cobalt and chromium levels in 1,900 patients with metal-on-metal hip arthroplasty
Source: Acta Orthop. 2020 Oct 2;91(6):711–6. doi: 10.1080/17453674.2020.1827191 (PMC8023953; doi:10.1080/17453674.2020.1827191)
Supplement: Supplemental Material [file IORT_A_1827191_SM6341.pdf]

Supplementary data

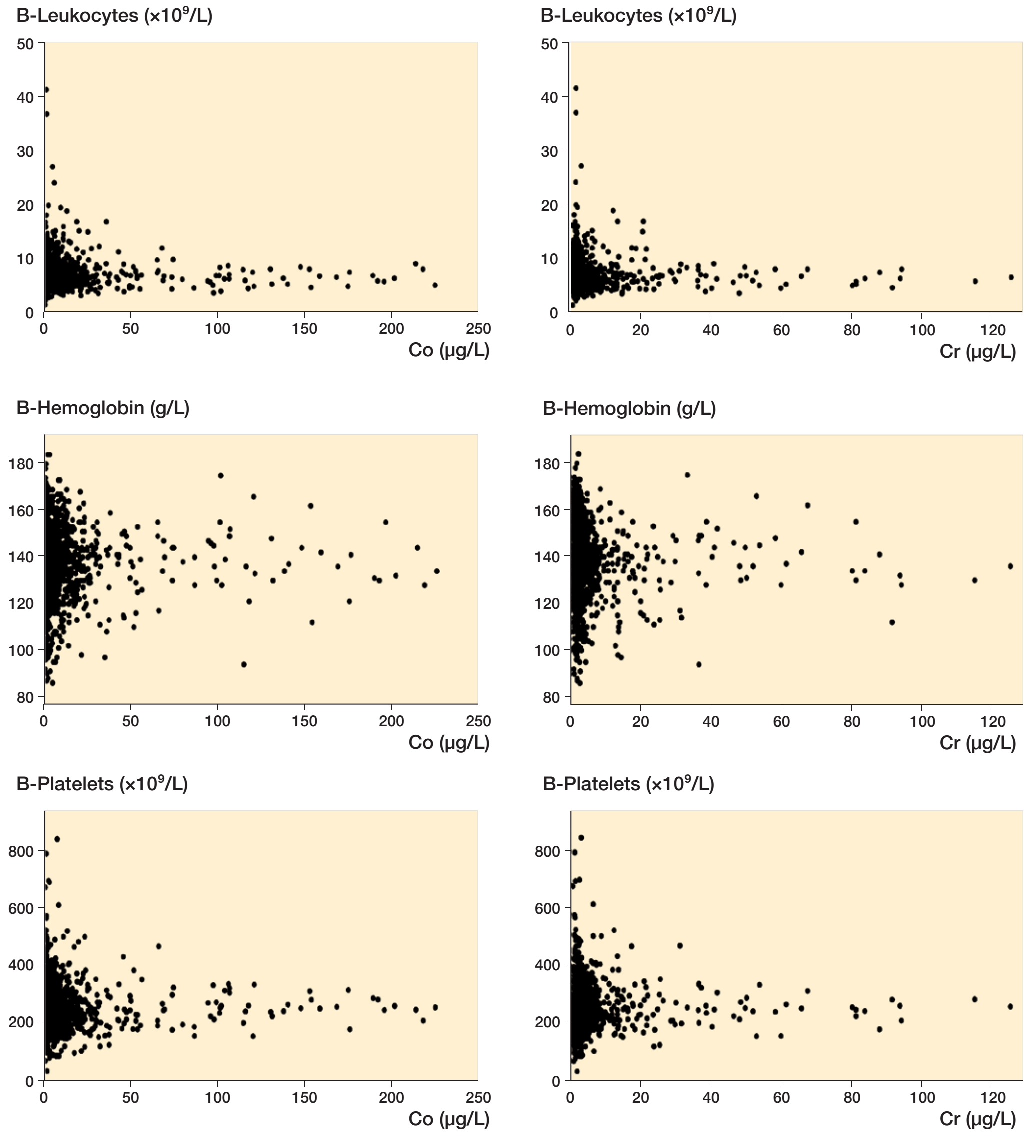

Figure 1. Scatter plot diagrams of relations between whole blood cobalt (Co) and chromium (Cr) levels and blood hemoglobin concentration, leukocyte and platelet counts.

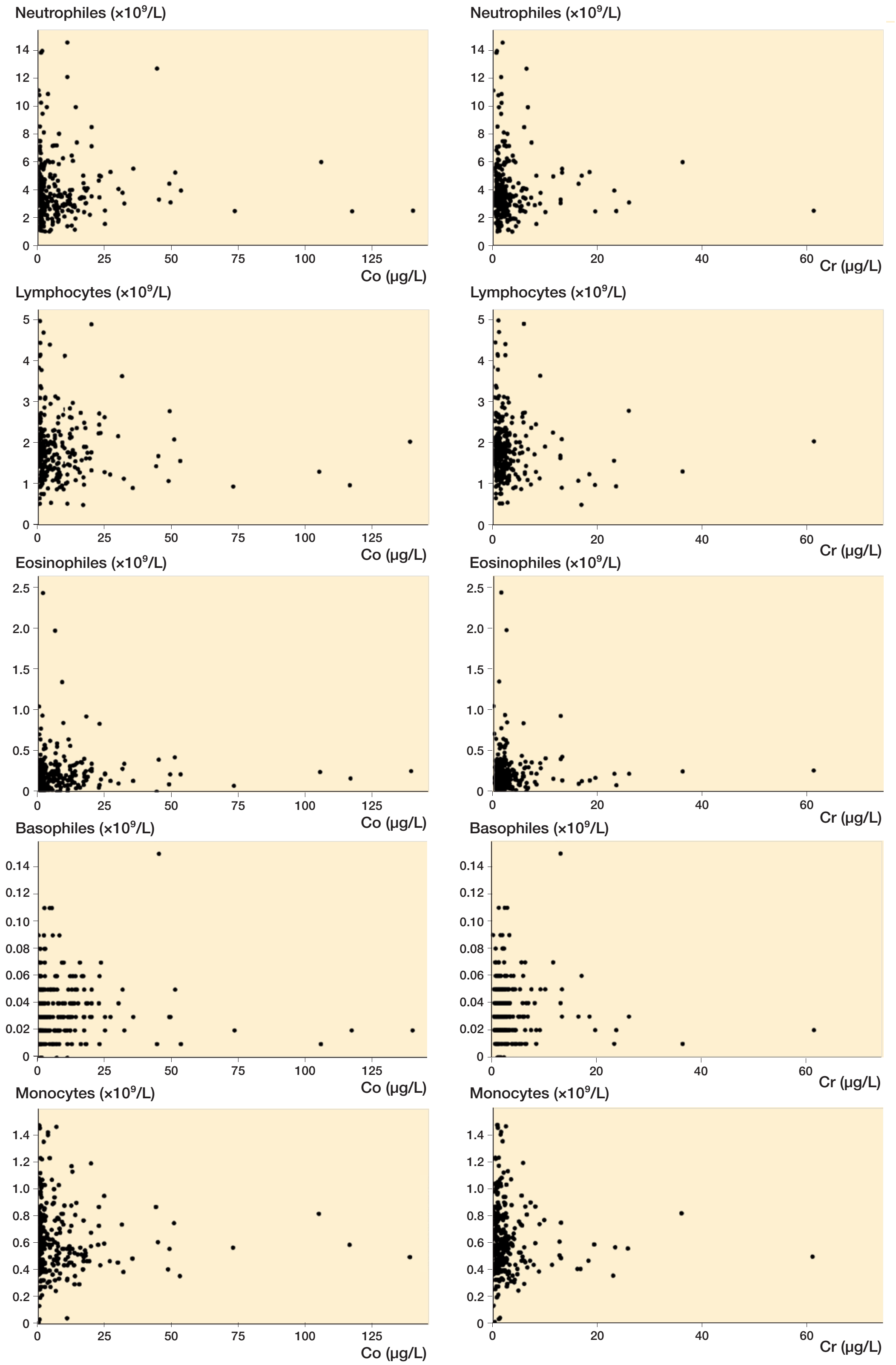

Figure 2. Scatter-plot diagrams of relations between whole blood cobalt (Co) and chromium (Cr) levels and leukocyte differentials counts.
